# Canopy nitrogen addition enhance the photosynthetic rate of canopy species by improving leaf hydraulic conductivity in a subtropical forest

**DOI:** 10.3389/fpls.2022.942851

**Published:** 2022-08-05

**Authors:** Guilin Wu, Dexiang Chen, Zhang Zhou, Qing Ye, Jianhui Wu

**Affiliations:** ^1^Hainan Jianfengling Forest Ecosystem National Field Science Observation and Research Station, Research Institute of Tropical Forestry, Chinese Academy of Forestry, Guangzhou, China; ^2^Key Laboratory of Vegetation Restoration and Management of Degraded Ecosystems, South China Botanical Garden, Chinese Academy of Sciences, Guangzhou, China

**Keywords:** canopy N addition, photosynthetic rate, leaf hydraulic conductivity, CO_2_ transportation distance, understory N addition

## Abstract

Elucidating the effects of atmospheric nitrogen (N) deposition on the photosynthetic capacity of plants is critical to understand forest growth and conservation under global change. However, studies on this topic generally consider only understory N addition, which ignores the effect of canopy interception. In this study, we conducted a field experiment in a subtropical forest to compare the effects of canopy vs. understory N addition on the photosynthetic rate of canopy and understory species. We found that canopy N addition enhanced the photosynthetic rate of canopy species by increasing leaf hydraulic conductivity and shortening the distance of CO_2_ transportation. In contrast, understory N addition had non-significant effects on the photosynthetic rate of canopy species. Moreover, the photosynthetic rate of understory species was not affected by canopy or understory N addition. Interestingly, changes in hydraulic conductivity contributed more to accelerating the photosynthetic rate than changes in CO_2_ transport distance. Our results provide important insights into the dissimilar effects of canopy and understory N addition on the photosynthetic rates of species in subtropical forests. Based on our findings, we highlighted the urgent need to consider canopy processes in future studies on N deposition.

## Introduction

Nitrogen (N) deposition has increased rapidly in recent decades (Vitousek et al., 1997; Galloway et al., 2004, 2008), and this has greatly impacted plant growth and forest community structures (Swaine, 1996; Zalamea et al., 2016). The physiological features of plants can reflect fundamental processes and the ecological strategies of plants in response to global change (Mao et al., 2017; Wu et al., 2018). Therefore, exploring the physiological mechanisms of how plants adapt to N deposition is fundamental for promoting species growth and conservation (Mo et al., 2008; Simkin et al., 2016).

Photosynthesis is the process by which chloroplasts use CO_2_ and H_2_O to synthesize starch. The photosynthetic capacity of plants is collectively governed by suites of traits (Liang et al., 2020). Among these, leaf hydraulic conductivity, stomatal traits, and leaf thickness are critical regulators of photosynthetic rate. Leaf water conductivity represents the capacity of leaves to transport H_2_O for the photosynthetic process and is associated with carbon accumulation in leaves (Brodribb et al., 2007). Stomatal traits and leaf thickness regulate the distance that CO_2_ has to travel to reach the chloroplast and may affect CO_2_ transportation to the site of photosynthesis (Noblin et al., 2008; Drake et al., 2019). A larger stomatal area, higher stomatal density, and lower leaf thickness can shorten the CO_2_ transport distance and accelerate the photosynthetic rate (Noblin et al., 2008; de Boer et al., 2012; Drake et al., 2019). Previous studies have generally explored the effects of N deposition on these traits individually (Liu N. et al., 2020; Zhang et al., 2021). To the best of our knowledge, no studies have explored the contributions of simultaneous changes in H_2_O conductivity and CO_2_ transportation on the photosynthetic rate of plants under N deposition.

Although most previous studies have focused on understory N addition, canopy N addition has received research attention in recent years (Zhang et al., 2015). Unlike understory N addition, canopy N addition takes into consideration the canopy interception process, which is closely associated with natural N deposition. Previous studies have reported that the canopy can intercept a large percentage of N in various types of forests, leading to contrasting nematode communities and differences in fine root biomass between forests with canopy vs. understory N addition (Gaige et al., 2007; Sievering et al., 2007; Liu T. et al., 2020; Li et al., 2021; Wang et al., 2021). If the intercepted N was absorbed by leaves (as suggested by previous studies), it would subsequently affect photosynthesis. However, few studies have explored the underlying mechanism of photosynthesis in species under different N addition treatments (Liu N. et al., 2020; Wang et al., 2021).

In this study, we selected four co-occurring dominant species, including two canopy trees (*Castanopsis chinensis* and *Schima superba*) and two understory species (*Ardisia quinquegona* and *Blastus cochinchinensis*). Photosynthetic rate, leaf hydraulic conductivity, leaf thickness, and traits related to stomatal anatomy (including stomatal area and stomatal density) were measured in the four species. We addressed the following questions: (1) how does canopy vs. understory N addition affect the leaf hydraulic conductivity, leaf thickness, and stomatal anatomy of canopy trees and understory species? and (2) how do these changes affect plant photosynthetic capacity under N addition?

## Materials and methods

### Study site

The study site was located in a subtropical natural forest in the Shimentai National Nature Reserve (24°22′–24°31′ N, 113°05′–113°31′ E) of Guangdong Province, Southern China. The mean annual temperature of this region is 20.8°C, with the lowest in January (10.9°C) and the highest in July (28.9°C). The mean annual precipitation is c. 2,300 mm, with 80% of the precipitation occurring during the wet season (June–October). The atmospheric wet N deposition rate in Guangdong province is higher (33.1 kg N/ha/year) than the global average (c. 5.2 kg N/ha/year; Peñuelas et al., 2013), and the soil type is latosolic red clay loam. The age of the broadleaved evergreen forest in Shimentai National Nature Reserve is more than 50 years (thinned in 1965), and the stand density is approximately 818 trees/ha.

### Experimental design and treatments

**FIGURE 1:**
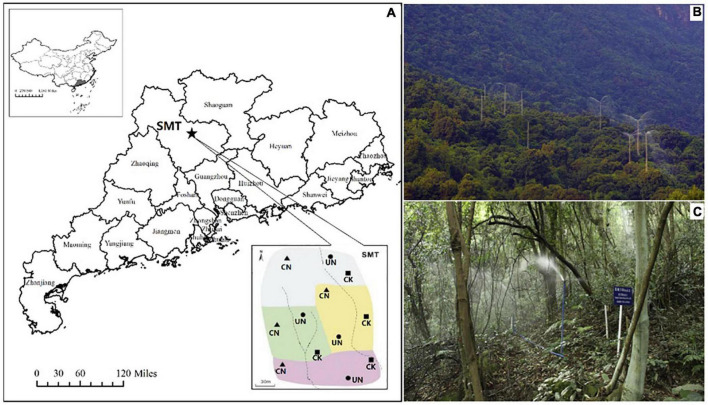
Geographical location and plot layout of the experiment **(A)** and the setups for canopy **(B)** and understory **(C)** N addition. CK, CN, and UN represent control conditions, canopy N addition, and understory N addition, respectively. Pictures of **(B,C)** were taken by Liu N. et al. (2020).

Both canopy (CN) and understory (UN) N addition treatments were designed with a rate of 50 kg N/ha/year. We also set a blank treatment (CK) with an N addition rate of 0 kg N/ha/year. There were four experimental blocks in this study, with each block including three randomly distributed plots (CK, CN, and UN; Figure 1A). The plots were 17 m in diameter and were separated by buffer zones at least 20 m wide. Polyvinyl-chloride boards were inserted to separate the buffer zones. Each plot occupied a 907 m^2^ area; of this, 400 m^2^ was the core sampling area, and the remaining area was designated as the buffer zone. The canopy N-addition treatments were achieved *via* a forest canopy spraying system (height, 35 m) built at the center of each plot. The understory N-addition system consisted of five sprinklers that were evenly distributed in each plot (height, 1.5 m). The sprinklers rotated 360° automatically at a constant speed to ensure uniform spraying (Figures 1B,C). Appropriate amounts of N were mixed to the approximate concentration of NH_4_NO_3_ in surface water obtained from an upper-position pond. Starting in 2013, the target N solution was sprinkled (rate, 3 mm precipitation per application) during April–October (seven times a year). The total amount of water sprayed was equivalent to 21 mm precipitation, accounting for less than 1% of the total rainfall of a year in the reserve. Therefore, the effects of water addition were negligible (Zhang et al., 2015; Lu et al., 2021). After 4 years of continuous N addition, we conducted our experiments during August and September of 2017.

Four dominant species, including two canopy tree species (*C. chinensis* and *S. superba*) and two understory species (*A. quinquegona* and *B. cochinchinensis*), were selected in our study. Three replicates were installed in each plot, and a total of 12 samples were collected from each treatment group.

### Measurement of gas exchange

Leaf gas exchange was measured on clear days in August and September of 2017 at 9:00–11:00 am using a portable photosynthesis system equipped with a CO_2_ injector (Li6800, Li-Cor, Lincoln, NE, United States). Each species was measured three times, and the photosynthetic rate was determined for five sun-exposed mature leaves from each individual plant. For understory species (*A. quinquegona* and *B. cochinchinensis*), we measured the gas exchange in plants without cutting the branches. For the canopy tree species (*C. chinensis* and *S. superba*), we cut the sun-exposed branches, placed the stem immediately in water, and then quickly measured the gas exchange. Based on preliminary trials, the photosynthetic photon flux density was set at 1,500 mol/m^2^/s to ensure that light-saturated photosynthetic rates were measured for all species. Ambient CO_2_ was maintained at 400 mol/mol, and leaf temperature was maintained at 26°C for all measurements. To avoid the impact of vapor pressure deficit on gas exchange, we also controlled the relative humidity in the chamber at 70–90% during the measurements. Before the data were recorded, the leaves were exposed to the above conditions for approximately 5–10 min to allow the photosynthetic parameters to stabilize.

### Measurement of leaf hydraulic conductivity

Leaf hydraulic conductance (*K*_leaf_) was determined using the method described by Brodribb and Holbrook (2003). Leaf-bearing canopy branches were collected at predawn and placed in a plastic black bag with moist towels for approximately 1 h to ensure that all attached leaves were at a similar water potential. The initial leaf water potential (ψ_leaf_) of equilibrated branches was determined using a pressure chamber (PMS Instruments, Corvallis, OR, United States), and the neighboring leaves were cut under water and allowed to rehydrate for 10 s. The water potential of the rehydrated leaves was measured immediately Zhang et al. (2021). Leaf hydraulic conductance was calculated as follows:


K=leafC×ln(ψ/0ψ)f/t

where *C* is the leaf capacitance, ψ_0_ is the leaf water potential before rehydration, ψ_f_ is the leaf water potential after rehydration for *t* seconds, and *t* is 10 s in our study. *C*-values pre- and post-turgor loss were calculated from the slopes of leaf pressure–volume relationships and were expressed in absolute terms normalized by leaf area:


C=ΔRWC/Δψ×1(DM/LA)×(WM/DM)/M

where RWC is the relative water content, DM is the leaf dry mass (g), LA is the leaf area (m^2^), WM is the mass of leaf water at 100% RWC (g), and M is the molar mass of water (g/mol).

### Measurement of stomatal traits and leaf thickness

On sunny days between 9:00 and 11:00 a.m., mature and sun-exposed leaves were collected in a transparent plastic bag with a moist towel and immediately transported to the laboratory. Stomatal traits were measured using the method described by Carins Murphy et al. (2012). In brief, the cuticles were stained with diluted (c. 0.1%) aqueous toluidine blue, rinsed, mounted on microscope slides, covered with a cover slip, gently pressured with fine-point tweezers, and then immediately observed under a light microscope (YS100, Nikon, Tokyo, Japan). Stomatal density (*SD*) and the width (W) and length (L) of guard cells were measured using image analysis software (OPTPro 2012 4.0, Optec XTS20, Chongqing Optec Instrument Co. Ltd., China). Stomatal area (*SA*) was defined as guard length (L) multiplied by the total width (2W) of the two guard cells (Franks et al., 2009).

The base, mid, and tip of each leaf were cut to obtain cross-sections. The sections were photographed with an Optec upright microscope (Chongqing Optec Instrument Co. Ltd.) equipped with a digital camera. The thickness of each section was determined using a computerized image analysis system (OPTPro 2012 version 4.0; Optec Software).

### Statistical analysis

Trait values were compared among the CK and N-addition treatments using nested ANOVA. To determine the mechanism of changes in photosynthetic rate under CN and UN conditions, we calculated the increments in physiological variables in N addition treatments (CN or UN) minus those in blank treatments (CK) for each species. We first analyzed the relationship between increments in photosynthetic rate (Δ*A*_n_) and the other four variables (i.e., Δ*K*_leaf_, Δ*LT*, Δ*SA*, and Δ*SD*) under N deposition using linear regression analysis. We then used the “varpart” function in the R package *vegan* v.2.5-6 (Oksanen et al., 2019) to estimate the unique contributions of Δ*K*_leaf_, Δ*LT*, and Δ*SS* (including Δ*SA* and Δ*SD*) to Δ*A*_n_. All analyses were conducted, and figures were prepared using RStudio version 4.0.3 (R Studio-Team, 2016).

## Results

### Response of leaf functional traits to canopy vs. understory nitrogen addition

In the two canopy tree species (*C. chinensis* and *S. superba*), the photosynthetic rate (*A*_n_), leaf hydraulic conductivity (*K*_leaf_), and stomatal area (*SA*) were significantly higher in CN (canopy N addition) than in CK (control). In contrast, leaf thickness (*LT*) was significantly lower under CN than under CK. The stomatal density (*SD*) of *S. superba* was significantly lower in CN than in CK, but the difference was non-significant in *C. chinensis*. In the understory species (*A. quinquegona* and *B. cochinchinensis*), there were no significant differences in *A*_n_, *K*_leaf_, *SA*, *LT*, *SA*, and *SD* between the CN and CK treatments. However, *A*_n_, *K*_leaf_, and *SA* tended to increase (less than 10%) under the CN treatment.

**FIGURE 2:**
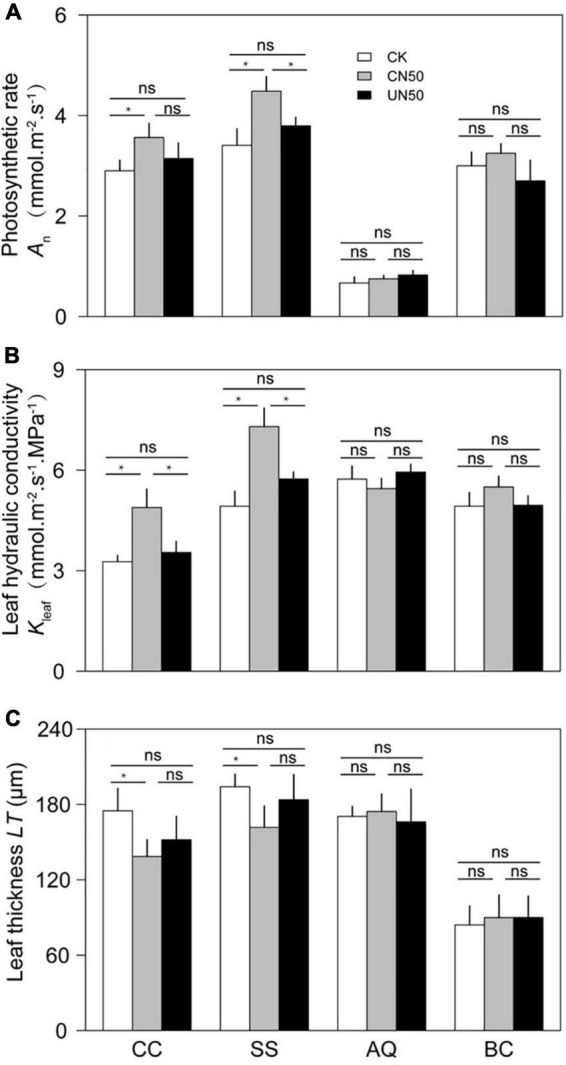
Photosynthetic rate **(A)**, leaf hydraulic conductivity **(B)**, and leaf thickness **(C)** of two canopy tree species (*CC* and *SS*) and two understory species (*AQ* and *BC*) under control conditions (CK), canopy N addition (CN), and understory N addition (UN). *CC*, *SS*, *AQ*, and *BC* are *Castanopsis chinensis*, *Schima superba*, *Ardisia quinquegona*, and *Blastus cochinchinensis*, respectively. ns: no significant difference; *Significant difference at *P* < 0.05 (nested ANOVA).

**FIGURE 3:**
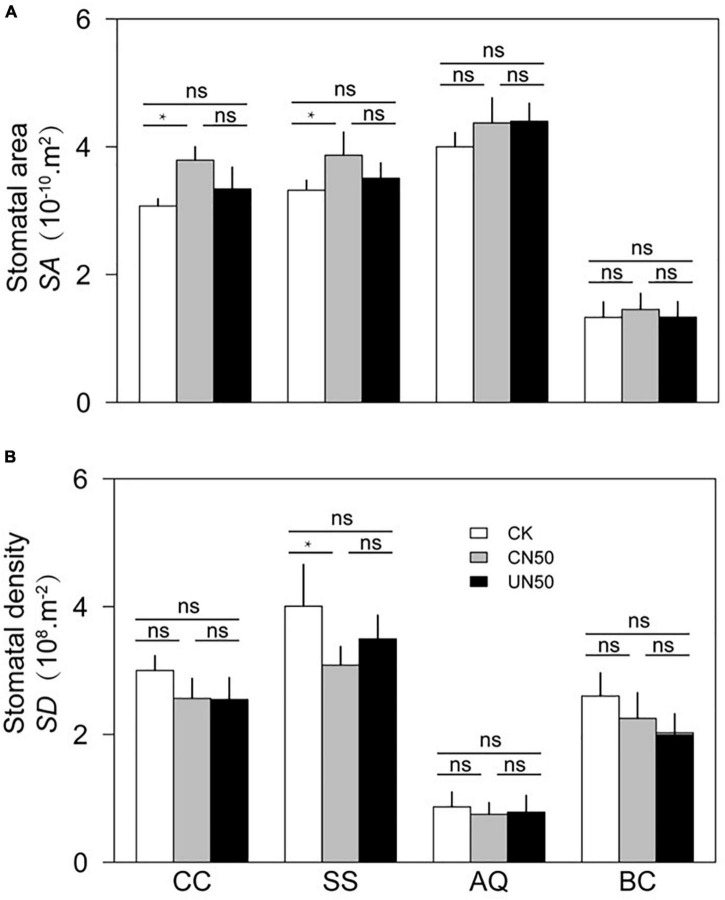
Stomatal area **(A)** and stomatal density **(B)** of two canopy tree species (*CC* and *SS*) and two understory species (*AQ* and *BC*) under control conditions (CK), canopy N addition (CN), and understory N addition (UN). *CC*, *SS*, *AQ*, and *BC* are *Castanopsis chinensis*, *Schima superba*, *Ardisia quinquegona*, and *Blastus cochinchinensis*, respectively. ns: no significant difference; *Significant difference at *P* < 0.05 (nested ANOVA).

The leaf functional traits (*A*_n_, *K*_leaf_, *SA*, *LT*, *SA*, and *SD*) did not differ significantly between UN and CK in any of the four species (Figures 2, 3).

### Comparison of leaf functional traits in canopy vs. understory nitrogen addition

In canopy species, the values of leaf functional traits in UN were generally intermediate between those in CK and CN. The *A*_n_ of *C. chinensis* differed between CN and UN, but the difference was not statistically significant. However, the *A*_n_ of *S. superba* was significantly higher under CN than under UN. The *K*_leaf_ values of *C. chinensis* and *S. superba* differed significantly between CN and UN. In contrast, *LT*, *SA*, and *SD* were not significantly different between CN and UN for either species (Figures 2, 3). The *A*_n_, *K*_leaf_, *SA*, *LT*, *SA*, and *SD* values of the two understory species did not differ significantly between CN and UN (Figures 2, 3).

### Associations between functional traits

**FIGURE 4:**
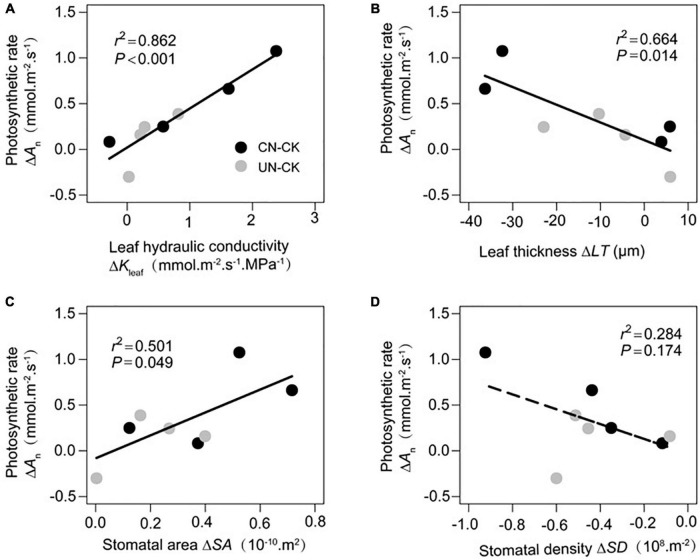
Relationship between increments in photosynthetic rate (Δ*A*_n_) and increments in leaf hydraulic conductivity (Δ*K*_leaf_, **A**), leaf thickness (Δ*LT*, **B**), stomatal area (ΔS*A*, **C**), and stomatal density (Δ*SD*, **D**). Gray and black circles represent CN minus CK and UN minus CK, respectively.

Increments in *A*_n_ (Δ*A*_n_) were significantly correlated with increments in *K*_leaf_ (Δ*K*_leaf_, Figure 4A) and *SA* (Δ*SA*, Figure 4C) and decrements in *LT* (Δ*LT*, Figure 4B). However, Δ*A*_n_ was not significantly associated with Δ*SD* (Figure 4D).

**FIGURE 5:**
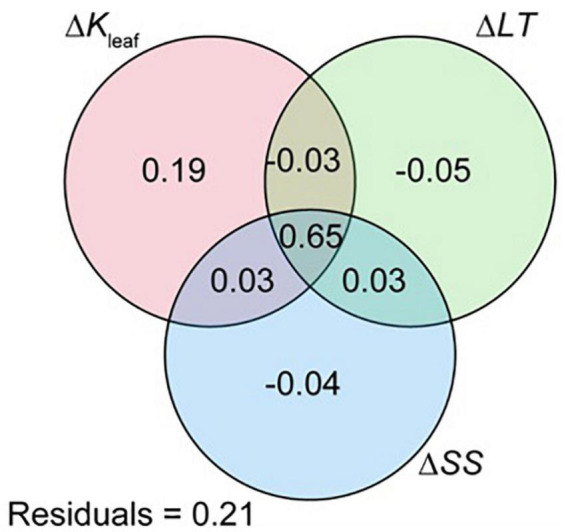
Venn diagram showing the results of variance partitioning on a full model of Δ*A*_n_ with Δ*K*_leaf_, Δ*LT*, and Δ*SS* (including Δ*SA* and Δ*SD*) as explanatory variables. Results are shown as a percentage of explained variance. *A*_n_, *K*_leaf_, *LT*, S*A*, and *SD* indicate photosynthetic rate, leaf hydraulic conductivity, leaf thickness, stomatal area, and stomatal density, respectively.

To identify which leaf functional traits contributed highly to increments in photosynthetic capacity, we estimated the individual effects of Δ*K*_leaf_, Δ*LT*, and Δ*SS* (Δ*SA* and Δ*SD*) on Δ*A*_n_. The unique effect of Δ*K*_leaf_ on Δ*A*_n_ was 19%, whereas those of Δ*LT* and Δ*SS* were almost negligible (<5%; Figure 5).

## Discussion

### Effect of nitrogen addition approaches on plant photosynthetic rate

Nitrogen is an important nutrient for plant growth. Most previous studies have explored the adaptive strategies of plants using understory N addition, which does not account for the canopy interception process. In this study, we compared photosynthetic rates and leaf hydraulic traits between canopy and understory N addition treatments. As expected, our results revealed contrasting patterns of change in photosynthetic rate among canopy species under different N addition treatments. These findings suggest that the effects of atmospheric N deposition on growth in forest ecosystems may be incorrectly estimated using the traditional UN method. A recent meta-analysis by Liang et al. (2020) indicated that changes in photosynthetic rate in response to N deposition varied among species and depended on the N deposition rate, duration of N deposition, species type, and climatic factors. Our study fills the knowledge gap regarding the effects of different N addition approaches on the photosynthetic rates of different species.

Consistent with previous studies (Zhang et al., 2018; Liu et al., 2021; Song et al., 2022), we found that the photosynthetic rate of canopy species was enhanced under canopy N addition. This can be explained by two reasons. First, increased leaf hydraulic conductivity accelerates the photosynthetic rate because CO_2_ accumulation is associated with water transportation efficiency (Brodribb et al., 2007; Zhu et al., 2013). Second, a larger stomatal area and lower leaf thickness under conditions of canopy N addition can shorten the CO_2_ transport distance (Noblin et al., 2008; Drake et al., 2019), thereby enhancing the photosynthetic rate. Unlike canopy N addition, understory N addition did not have large effects on the leaf functional traits (including photosynthesis rate, leaf thickness, and stomatal traits) of canopy species. These discrepant responses to understory and canopy N addition may be because canopy leaves absorbed N directly, which immediately accelerated their photosynthetic rate (Chiwa et al., 2012; Wang et al., 2021). In contrast, understory N addition may have only affected the nutrient concentration in the soil. Since subtropical forests in southern China are generally N-rich (Tian et al., 2018), N addition in soil may not affect photosynthesis in canopy leaves. Furthermore, southern China experiences high precipitation, especially in summer (mean annual precipitation, 1,900 mm; 80% in summer). As such, dissolved inorganic N leaching after frequent precipitation would dilute the additional N in the soil. An alternative explanation is that soil N takes longer to be transported to the leaves, which may cause a lag between soil N addition and responses in leaf metabolism. For example, Wang et al. (2021) reported that the photosynthetic rate of aspen saplings increased after 15 days following canopy N addition and after 20 days following understory N addition. Therefore, future studies should conduct long-term and continuous monitoring of photosynthetic rates in plants under understory and canopy N addition treatments.

Somewhat unexpectedly, the photosynthetic rate of understory species did not change significantly under either canopy or understory N addition, even though both approaches can add N to the leaves of understory species. One possible explanation is that subtropical forests are usually complex and dense, and several other factors may be affecting the photosynthetic rate of understory species. For example, the light intensity may play a critical role in regulating the photosynthetic capacity of understory species. Previous studies have indicated that N addition can increase the total leaf area (Liang et al., 2020) or the ratio of distal leaf area to sapwood area (Fang et al., 2018). These changes in the leaves of canopy species would increase the level of shade on the forest floor, which would negatively affect the photosynthetic rate of understory species. This may offset the positive effects of N addition on the leaves of understory species. However, understory N addition has been shown to significantly enhance the photosynthetic capacity of an understory species (*Psychotria rubra*) in a subtropical forest Liu N. et al., 2020), which is inconsistent with our results. It is possible that adaptation strategies and sensitivity to N addition differ among plant species, as suggested by Liang et al. (2020). Therefore, more species should be included in future studies.

### Effect of changes in H_2_O and CO_2_ transport capacity on photosynthetic rate

In all species under canopy and understory N addition treatments, increments in photosynthetic rate were significantly correlated with variations in leaf water conductivity and CO_2_ transport distance (*ΔLT* and *ΔSS*). This indicates that the simultaneous adjustment of H_2_O and CO_2_ transport capacity due to N addition can enhance leaf photosynthetic capacity. Most previous studies have explored the relationship between leaf hydraulic traits and photosynthetic rate (Brodribb et al., 2007; Zhang and Cao, 2009; Zhu et al., 2013) or have compared leaf hydraulic structures and anatomic traits under different environments (Wu et al., 2018; Zhang et al., 2021). Although the results suggested that hydraulic traits and C accumulation are coupled, few studies have explored the patterns of variation in these traits under changing environments. This study improves our understanding of this association by showing that variations in these traits are also coupled under nitrogen addition.

Interestingly, we found that compared with increments in CO_2_ transport ability, increments in leaf hydraulic conductivity had a much higher unique contribution to the acceleration of photosynthetic rate under N addition treatment in a subtropical forest. Schneider et al. (2017) reported that in Ochnaceae species, shortening the CO_2_ transport distance by improving stomatal anatomic traits was more economical than adjusting the water transport structure (e.g., by increasing the density of minor veins). One possible explanation for our results is that adjusting water transport-related traits is more “expensive” for plants than changing CO_2_ transport-related traits, leading to insufficient adjustment of leaf hydraulic conductivity. As a result, the leaf water transport capacity is not optimized under N addition, and becomes a bottleneck to increments in photosynthetic rate. We also found that the 21% change in photosynthetic rate could not be explained by changes in leaf functional traits in our study. It is possible that other traits (such as chlorophyll content) have additional effects on photosynthetic rate, as indicated in previous studies (Barroco et al., 2006; Lehmeier et al., 2017; Zeng et al., 2022). Therefore, more plant traits should be included as variables in future studies.

## Conclusion

Our results indicate that canopy and understory N addition have contrasting effects on the photosynthetic rate of canopy trees in a subtropical forest in southern China. Canopy interception of N enhanced the photosynthetic rate of canopy tree species by increasing leaf hydraulic conductivity and reducing the CO_2_ transport distance. In contrast, the photosynthetic rates of understory species were not affected under canopy or understory N addition. We also found that compared with changes in CO_2_ transport distance, changes in hydraulic conductivity contributed more to enhancing the photosynthetic rate. Our results provide important insights into the contrasting effects of canopy vs. understory N addition and highlight the urgent need to consider canopy processes in future studies on N deposition.

## Data availability statement

The original contributions presented in the study are included in the article/Supplementary material, further inquiries can be directed to the corresponding author.

## Author contributions

DC and GW conceived the idea. GW performed the analyses and drafted the manuscript. GW, DC, ZZ, QY, and JW contributed substantially to the revisions. All authors have read and agreed to the published version of the manuscript.
